# Haematopoietic Stem Cell Transplantation and the Price to Pay For

**Published:** 2013-01-04

**Authors:** N. Maffulli

**Affiliations:** University of Salerno

Haematology/oncology and trauma and orthopaedics do marry. The well thought out article by the group led by Prof. Selleri [1] elegantly points up what, empirically, some of us already knew: haematopoietic stem cell transplantation does save lives and prolongs survival, but the price to pay, for the musculoskeletal system, can be high. Bone is a composite tissue, of which its haematopoietic component is a major part. The societal impact of autologous and allogeneic haematopoietic stem cell transplantation for the management of malignant and non-malignant haematological conditions has now become common place, and Salerno is a leading centre in the Mediterrean and Europe in this field. Nevertheless, there is a price to pay: it is possible that, by depriving bone of ‘its own’ haematopoietic bone marrow, the result is that the local metabolism is altered. This, coupled with the vast array of drugs that these patients receive, may well result in the deleterious effects on the musculoskeletal system that are described in this article. The association between corticosteroids and avascular necrosis of the femoral head is well described, but, if it is associated with osteoporosis, the resulting combination could well be very difficult to manage: any reconstructive procedures may be destined to fail.

In addition, although not reported in this investigation, it is likely that the levels of activities of such patients may well drop following these taxing treatments, and that the cocktail of drugs and the transplant themselves may have noxious effect on muscles, with sarcopenia: the effects on bone tissue are disastrous.

Monitoring bone mineral density using ultrasonography or DEXA is feasible, but the costs of such programmes should be put in the context of never ending cuts to the National Health Service. One should ask whether they satisfy the central tenets of screening: even though we can identify the amount of bone loss and we can prospectively monitor it, is it correctable, and will it make a clinically relevant difference? The authors rightly point out that bisphosponates (the use of which is already frowned upon in some orthopaedic and rheumatological circles) are badly tolerated in recipients of haematopoietic stem cell transplantation. It may be that, contrary to what big pharma wants us to believe, the best approach is to introduce controlled regular physical exercises [2]: the evidence in other medical fields is that exercise is medicine!
